# Impact of gelatin supplementation on indices of skeletal muscle recovery: a pilot study

**DOI:** 10.1007/s00421-026-06288-5

**Published:** 2026-06-02

**Authors:** Rebecca R. Graham, Brian E. Salgado, Emedel Guerrero, Ana P. Carrasco, Andrew C. D’Lugos

**Affiliations:** 1 Department of Kinesiology, James Madison University, 261 Bluestone Drive, Harrisonburg, VA 22807 USA; 2 Department of Kinesiology, California State University, Chico, Chico, CA USA

**Keywords:** Collagen, Extracellular matrix, Muscle damage, Eccentric exercise

## Abstract

**Purpose:**

This pilot study examined whether collagen supplementation, via gelatin, influences indices of skeletal muscle recovery following strenuous exercise.

**Methods:**

Using a double-blind, randomized, placebo-controlled design, recreationally-active adults (25.9 ± 4.6 yrs.; 11 F, 11 M) consumed gelatin (GEL; 15 g) or placebo (PLA) twice daily. Participants performed 150 maximal unilateral eccentric knee extensions. Muscle soreness (MS), assessed passively and dynamically (concentric, eccentric), peak isokinetic (PKT180, PKT60) and isometric torque (PMT), muscle morphology, and serum hydroxyproline (HYP) and procollagen-1 N-terminal peptide (P1NP) were assessed prior to- (Pre), 24 h, 72 h, and 168 h post-exercise.

**Reports:**

Exercise significantly increased soreness and impaired function over time (soreness: partial-eta-squared=0.56–0.76; torque: partial-eta-squared=0.34–0.61; all *P*<0.001), while group and group by time effects were not significant for these outcomes (*P*>0.05). Exploratory, pairwise comparisons suggested lower MS in GEL at select timepoints, including lower concentric MS at 24 h (*P*=0.029, d=0.97) and lower passive MS at 168 h (*P* = 0.028, d = 0.99). Differences in contractile function between groups were not statistically significant, though exploratory estimates favored GEL at 72 h for PKT60 (*P* = 0.067, d = 0.80) and PMT (*P* = 0.066, d = 0.81). HYP increased over time (partial-eta-squared=0.47, *P*<0.001) without group differences, and P1NP did not change over time or differ by group (*P*>0.05).

**Conclusion:**

*GEL* was associated with moderate-to-large time-specific effect estimates suggesting lower soreness and potentially improved force recovery at select timepoints; however, no significant group by time interactions were detected. These findings should be interpreted as preliminary and hypothesis-generating, supporting future trials into the effects of GEL as a recovery modality.

**Supplementary Information:**

The online version contains supplementary material available at 10.1007/s00421-026-06288-5.

## Introduction

Skeletal muscle is fundamental for performing a wide range of activities, ranging from those associated with sport (i.e., running, jumping) to those required for daily living (i.e., breathing, postural stability). Exercise is a potent stimulus for improving skeletal muscle health, and therefore overall health. Indeed, skeletal muscle is regarded for possessing a remarkable capacity to adapt to exercise stimuli. However, strenuous exercise can result in ultrastructural damage to skeletal muscle (Hyldahl and Hubal 2014; Mackey and Kjaer 2017a, b). This phenomenon is often referred to as exercise-induced muscle damage (EIMD) and is characterized by a variety of symptoms including impaired contractile function (Goodall and Howatson 2008; Brown et al. 1997a; Mackey et al. 2008; Neltner et al. 2023), soreness (Cheung et al. 2003; Byrne et al. 2004; Neltner et al. 2023), and inflammation (Howatson and van Someren 2008; Peake et al. 2017). These symptoms can negatively affect exercise performance and in some cases impede activities of daily living (Dannecker and Koltyn 2014).

While a majority of research into the mechanisms of EIMD have largely focused on damage to the muscle fibers (Mackey and Kjaer 2017b; Mackey et al. 2008), an often overlooked component of skeletal muscle is the extracellular matrix (ECM) in which muscle fibers are embedded (Csapo et al. 2020). The ECM is comprised of a network of collagens, glycoproteins and polysaccharide molecules creating a complex meshwork that encase the muscle fibers (Thorsteinsdóttir et al. 2011; Takala and Virtanen 2000). This scaffolding is crucial in providing structural integrity to muscle and transmitting forces throughout the muscle to facilitate whole-muscle contraction and movement (Mackey et al. 2011; Hyldahl and Hubal 2014; Gillies and Lieber 2011). In addition to its role as a structural scaffold, the ECM also orchestrates complex interactions between the many cell types that reside within skeletal muscle (i.e., satellite cells, immune cells, endothelial cells) and govern muscle adaptation to exercise (Thorsteinsdóttir et al. 2011; Daley et al. 2008; Rozario and DeSimone 2010). Furthermore, remodeling of the skeletal muscle ECM has also been demonstrated as a vital process underlying muscle hypertrophy (Fry et al. 2017; Hyldahl et al. 2015), supporting the role of the ECM in muscle health. Previous research has demonstrated damage to the skeletal muscle ECM in response to strenuous exercise, particularly eccentric exercise (Mackey et al. 2004; Miller et al. 2005; Hody et al. 2019; Brown et al. 1997b; Neltner et al. 2023), highlighting its susceptibility to EIMD. Given the many important functions of the skeletal muscle ECM, disruption to this structure is believed to play an important role in mediating the symptoms associated with EIMD (Mackey et al. 2011).

Following strenuous exercise, symptoms associated with EIMD can persist from days to weeks, as skeletal muscle undergoes repair and regeneration (Dupuy et al. 2018; Brown et al. 1997b). It is understood that repair of the ECM following EIMD is largely regulated by collagen synthesis (Moore et al. 2005). The remodeling of skeletal muscle ECM is often observed through increased circulating levels of collagen-specific amino acids, such as hydroxyproline (HYP), which may indicate collagen breakdown (Brown et al. 1997b; Tofas et al. 2008; Neltner et al. 2023), and procollagen 1 N-terminal peptide (P1NP), which reflects the synthesis of type-1 collagen (Kehlet et al. 2018; Shaw et al. 2017; Lee et al. 2023). Impairments to the repair and remodeling of the ECM may lead to structural changes that compromise not only contractile function but may potentially contribute to musculoskeletal injuries (Damas et al. 2016; Krentz and Farthing 2010; Mackey et al. 2011). Thus, avenues to enhance the remodeling of the ECM following damaging exercise may expedite muscle recovery and augment subsequent adaptation to exercise.

Recently, dietary collagen supplementation has been investigated as a means to enhance the beneficial effects of exercise on aspects of musculoskeletal health (Shaw et al. 2017; Clifford et al. 2019; Prowting et al. 2021; Holwerda and van Loon 2022; Lee et al. 2023; Lopez et al. 2015; Khatri et al. 2021; Bischof et al. 2024; Nulty et al. 2024). Collagen, the most abundant protein in the human body and the principal component of the ECM, is crucial to the structural integrity of the ECM, and therefore skeletal muscle. Additionally, collagen provides an elastic property to the ECM responsible for transmitting contractile forces from the muscle fiber to the surrounding musculoskeletal structures (i.e., tendons and bones) (Khatri et al. 2021; Holwerda and van Loon 2022). Collagen supplementation can be administered in the form of collagen peptides or hydrolyzed collagen. Both routes of ingestion provide amino acids of collagen (i.e., glycine, proline, and hydroxyproline) that are required for the repair and remodeling of the ECM following exercise (Holwerda and van Loon 2022). Work by Clifford et al. (2019) demonstrated supplementation with collagen peptides was effective at attenuating muscle soreness and expediting the recovery of muscle function following EIMD (Clifford et al. 2019). However, compared to hydrolyzed collagen, collagen peptide supplements are typically more expensive, presenting a potential barrier for their widespread adoption. Hydrolyzed collagen can be obtained in the form of gelatin, a commercially-available and cost-effective product. Seminal work from the Baar lab demonstrated the ingestion of vitamin C-enriched gelatin (15 g) prior to exercise is capable of significantly enhancing collagen synthesis (Shaw et al. 2017), a finding that has been validated more recently (Nulty et al. 2024). This suggests that gelatin may be an affordable, readily available means to enhance ECM remodeling following strenuous exercise, thus expediting muscle recovery. Currently, the impact of collagen supplementation, via gelatin, on muscle recovery following strenuous exercise is unknown. Therefore, the aim of the current study is to examine the effects of collagen supplementation, via gelatin, on indices of skeletal muscle recovery following strenuous exercise, and generate preliminary effect estimates to inform future studies.

## Methods

### Subjects

Twenty-three healthy males and females volunteered for this study. All participants were considered recreationally-active (performing ≤ 2 resistance exercise sessions per week) and were not engaged in a regularly scheduled resistance exercise training program. Screening was performed using an online survey (Qualtrics), as well as a Physical Activity Readiness Questionnaire (PAR-Q). Participants were eligible for inclusion if they were between 18 and 40 years of age and had a body mass index (BMI) ≤ 30 kg/m^2^ (non-obese). Exclusion criteria included any acute or chronic illness, including but not limited to cardiac disease, uncontrolled hypertension, diabetes, or other metabolic disorders, arthritis of the knees or hips, a history of neuromuscular problems, lower limb injuries impeding function, pregnancy, or use of hormonal contraceptives. All subjects provided written informed consent prior to participation. One participant failed to complete the study due to reasons unrelated to the study procedures and thus 22 participants completed all aspects of the study (Table 1). All procedures were approved by the California State University, Chico Institutional Review Board (IRB#: IRB-2023-7), which is in compliance with the Declaration of Helsinki as revised in 2024.

### Experimental design

**Fig. 1:**
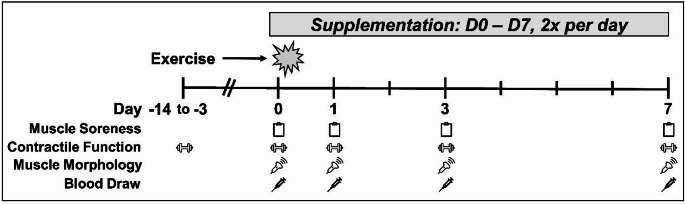
Schematic of experimental protocol. A familiarization session with the contractile function assessments and the eccentric exercise procedures occurred once between days − 14 to –3. Pre-exercise measures (PRE) occurred on day 0, followed immediately by an acute bout of damaging eccentric exercise. Participants returned to the laboratory for follow-up measures 1 (24 h), 3 (72 h), and 7 (168 h) days following the damaging exercise. Supplementation (GEL or PLA) occurred twice daily, beginning prior to the damaging exercise on Day 0 and continuing until Day 7

In a randomized, double-blind, placebo-controlled trial, participants were randomly assigned to one of the two supplement groups; an experimental group which received 30 g/day of gelatin (GEL; *n* = 10), and a control group which received an isovolumetric placebo (PLA; *n* = 12). Participants were randomly allocated to supplement groups using a computer-generated randomization schedule while striving to achieve a balanced sex distribution in each group. Participants completed a familiarization session consisting of unilateral knee extension exercise on an isokinetic dynamometer (System 4 Pro Biodex Medical Systems; Shirley, NY) using a self-selected limb. Three-to-fourteen days later, participants returned to the laboratory to complete one of four experimental visits (Fig. 1). During the first experimental visit, baseline (PRE) measures consisting of ultrasound imaging of the quadriceps of the selected leg, ratings of muscle soreness, peak isokinetic torque, and peak isometric torque were conducted prior to exercise. Following baseline measures, participants completed a single bout of muscle-damaging unilateral exercise. Participants returned to the laboratory 24 h, 72 h, and 168 h following the muscle-damaging exercise at which point baseline measures were repeated to assess the time course of muscle recovery.

### Procedures

#### Supplementation

Supplements were provided to participants in a double-blind fashion, and were shape-, texture-, color-, and taste-matched between the GEL and PLA groups. A dedicated member of the research team (A.C.), who did not partake in data analysis, assigned codes the supplement groups and prepared all supplements for participants. Supplements were provided in the form of four 2.5 cm x 2.5 cm x 1.3 cm chewable square gummies, delivered to participants in opaque plastic containers to take home. No formal assessment of blinding assessment was conducted; however, participants reported no awareness of group assignment. The formulation of the GEL supplements was adapted from Shaw et al. (2017). Supplements for the GEL group included 15 g of gelatin (Bovine Gelatin, Now Foods; Bloomingdale, IL), 50 mg vitamin C (Pure Manufacturing; Lindon, UT), 500 mg sucralose, 7.3 ml sugar-free strawberry flavoring (Torani; San Leandro, CA), and 25 ml water. Supplements for the PLA group included the exact same ingredients with 667 mg agar (Now Foods; Bloomingdale, IL) in place of gelatin. Participants were instructed to consume supplements twice per day, once in the morning and once in the evening, resulting in a daily GEL dose of 30 g/day. To ensure compliance with the dosing schedule, a member of the research team communicated with participants via email daily during the 7-d experimental time frame. On the days of experimental visits, participants were provided their morning dose of supplements 45 min prior to any muscle function testing (Shaw et al. 2017).

#### Dietary and exercise controls

Participants were instructed to abstain from any heavy or unaccustomed physical activity for 48 h prior to the first experimental visit. Throughout the 168 h follow-up period (experimental visits 2–5), participants were instructed to refrain from any additional exercise outside of the laboratory. On the morning of each experimental visit, participants arrived to the laboratory after a 10 h overnight fast and remained fasted until completion of the visit (water provided *ab libitum*). Participants were also instructed to refrain from alcohol consumption for 24 h before each experimental visit. Throughout the entire study, participants were instructed to maintain normal dietary habits, while abstaining from consuming any over-the-counter supplements, including vitamin C and collagen supplements, and any prescription or non-prescription anti-inflammatory or analgesic medications.

#### Muscle damaging protocol

The muscle damaging exercise bout was performed on the first experimental visit immediately following muscle contractile function testing (see below). The exercise consisted of 150 maximal unilateral eccentric knee extensions (10 sets of 15 reps, separated by 2 min of rest) on an isokinetic dynamometer using a self-selected limb. Participants received verbal encouragement and visual feedback of the force produced during each repetition to ensure maximal effort was attained. This protocol was adapted from previous studies that have demonstrated its ability to induce a high degree of localized skeletal muscle damage (Farup et al. 2014; Baumert et al. 2018; Mackey et al. 2004; Mikkelsen et al. 2011).

#### Muscle soreness

Muscle soreness (MS) of the quadriceps of the selected limb was assessed using a visual analog scale, where 0 mm indicated no muscle soreness and 100 mm indicated extreme soreness/stiffness (D’Lugos et al. 2016). Participants reported subjective muscle soreness in three contractile phases; passive (standing still), concentric (stepping up onto a 20 cm step), and eccentric (stepping down off of a 20 cm step). Muscle soreness was assessed prior to- and 24 h, 72 h, and 168 h after the damaging bout of exercise.

#### Muscle contractile function

Immediately following assessments of muscle soreness, participants completed a 5 min walking warm-up on a treadmill at 2.5 mph. Participants were fitted to an isokinetic dynamometer to assess peak isokinetic (PKT) and isometric torque (PMT) of the quadriceps of the selected limb. PKT was assessed at two contractile velocities; first at 180 deg∙sec^− 1^ (PKT180), followed by 60 deg∙sec^− 1^ (PKT60). For each contractile velocity participants completed two sets of three maximal unilateral knee extensions separated by 1 min of rest. For PMT, participants performed three, 3-second maximal isometric unilateral knee extensions at 60° knee flexion, separated by 1 min of rest. Muscle contractile function was assessed immediately prior to- and 24 h, 72 h, and 168 h after the damaging bout of exercise.

#### Muscle morphology

To assess tissue swelling, skeletal muscle thickness (MT) of the *vastus lateralis* (VL), *rectus femoris* (RF), and *vastus intermedius* (VI) of the selected limb were examined with two-dimensional (2D) brightness mode (B-mode) ultrasonography (LOGIQe, GE; Boston, MA) using a 4.7 cm linear array transducer (4–13 MHz) in the longitudinal plane (see Online Resource 1 for representative image). Settings of the focal points, dynamic range, gain, and frequency were held constant across all participants and time points. All imaging sites were identified based on bony landmarks of the femur, to assess morphology of the quadriceps at multiple anatomical locations, as previously described (Blazevich et al. 2006; D’Lugos et al. 2024). Imaging sites included a lateral and anterior location corresponding to 56% of the distance from the lateral condyle of the knee to the head of the greater trochanter. Imaging sites were denoted on the skin with a surgical-grade skin marker, which the participants used to preserve the markings between experimental visits.

Prior to imaging, participants rested in a seated posture on a medical examination table for 15 min to allow for any potential intramuscular fluid shifts to subside (Berg et al. 1993). A towel-roll was placed under the knee of the selected limb to achieve natural knee flexion of approximately 10°. Hip flexion and rotation were standardized for each participant across time. Muscle morphology was assessed prior to- and 24 h, 72 h, and 168 h after the damaging bout of exercise.

#### Ultrasound image analysis

Ultrasound images were analyzed for MT as previously described (D’Lugos et al. 2024). Image analysis was conducted using a publicly available software (ImageJ/Fiji, National Institutes of Health; USA). MT of the VL and RF was measured as the perpendicular distance between the border of subcutaneous fat and muscle to the deep aponeurosis. In the VI, MT was measured as the perpendicular distance between the superficial aponeurosis and the superficial border of the femur.

#### Circulating markers of collagen remodeling

Participants arrived at the laboratory for each experimental visit following a 10 h overnight fast and completed 10 min of rest in a phlebotomy chair prior to venipuncture. All blood samples were collected from an antecubital vein using a 21-guage needle and silica-coated vacutainers (367815, BD Biosciences, Franklin Lakes, NJ). Whole blood was allowed to clot at room temperature 30 min prior to centrifugation at 4 °C for 10 min at 2000 G to remove the serum portion. Serum was stored in a -80 °C freezer for subsequent analyses. Blood samples were collected prior to- and 24 h, 72 h, and 168 h after the damaging bout of exercise. Markers of collagen remodeling, including procollagen 1 N-terminal peptide (P1NP) and hydroxyproline (HYP), were measured using an enzyme-linked immunosorbent assay (ELISA; ab210966, Abcam, Cambridge, UK) and colorimetric assay (MAK008, Millipore Sigma, Saint Louis, MO), respectively, following manufacturer instructions.

### Statistical analyses

All data are expressed as means with individual datapoints unless denoted otherwise. All data were tested for normality prior to statistical analyses. Comparisons were performed using a two-way (group*time) ANOVA with repeated measures on the time factor (Pre, 24 h, 72 h, and 168 h post-exercise). Main effects and interactions, along with effect sizes (*partial eta square*, $\:{{\upeta\:}}_{\mathrm{p}}^{2}$) and 95% confidence intervals (CI) are reported for all primary outcomes measures. Due to the pilot nature of this study, exploratory LSD-adjusted pairwise comparisons were conducted to characterize patterns in the data and estimate effects for future studies. Effect sizes and 95% confidence intervals are reported as *Cohen’s d* for between-group comparisons and *Cohen’s d*_*z*_ for within-group comparisons. While the current study was not designed to test sex as a biological variable, exploratory analyses of biological sex as a moderating variable were performed using a three-way (group*time*sex) ANOVA with repeated measures on the time factor (Supplemental Figs. 2–4). Significance for all analyses was set a priori at *P* ≤ 0.05. All analyses were performed with GraphPad Prism Software (v.10.2.3, GraphPad Software; Boston, MA).

A priori sample size estimates were based on the most similar studies to date examining the effects of collagen supplementation on recovery following exercise (Prowting et al. 2021; Clifford et al. 2019; Kuwaba et al. 2023). Utilizing a range of previously reported effect sizes ranging from 0.50 to > 2.0 for outcomes measures including muscle soreness and contractile function, sample size estimates ranged from 12 to 26 participants per group to detect group differences in muscle soreness or contractile function with 80% statistical power at an alpha of 0.05. Given the pilot nature of this study and previously reported power analyses in similar investigations (Kuwaba et al. 2023; Prowting et al. 2021), a minimum sample size of *n* = 12 per group was targeted.

## Results

### Participant characteristics

Participant characteristics (age, height, weight, BMI) and baseline PMT are presented in Table 1. There were no group differences in any of these variables (*P* > 0.05).

### Muscle soreness

**Fig. 2:**
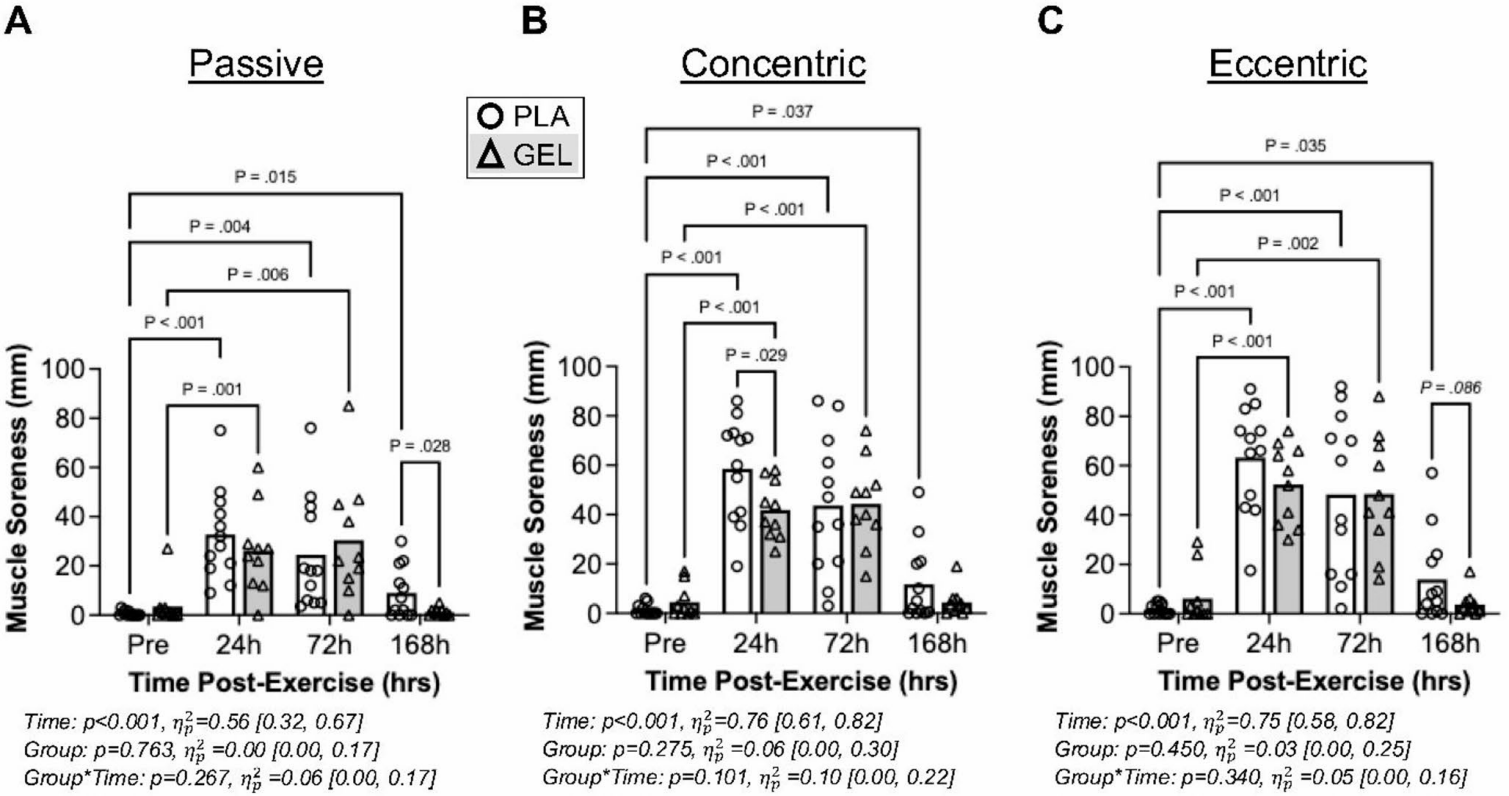
Subjective ratings of quadriceps muscle soreness (MS) prior to- and following damaging exercise. MS was assessed during three contractile phases: Passive (**A**), concentric (**B**), and eccentric (**C**). White bars and circles represent group averages and individual data for placebo (PLA; *n* = 12), respectively. Grey bars and triangles represent group averages and individual data for gelatin (GEL; *n* = 10), respectively

Pre-exercise ratings of static, concentric, and eccentric MS were not different between groups (*P* > 0.05, Fig. 2). For passive MS (Fig. 2A), there was a significant effect of time (*P* < 0.001, $\:{{\upeta\:}}_{\mathrm{p}}^{2}$=0.56 [95% CI *0.32*,* 0.67*]), but no significant effect of group (*P* > 0.05) or group by time interaction (*P* > 0.05). Exploratory pairwise comparisons within-group revealed increased passive MS in PLA at 24 h (*P* < 0.001, d = 1.8 [*1.2*,* 3.7*]), 72 h (*P* = 0.004, d = 1.1 [*0.49*,* 2.5*]), and 168 h (*P* = 0.015, d = 0.83 [*0.25*,* 2.0*]), and increased passive MS in GEL at only 24 h (*P* = 0.001, d = 1.5 [*0.79*,* 2.5*]), 72 h (*P* = 0.006, d = 1.1 [*0.60*,* 2.3*]). Between-group pairwise comparisons revealed a significant large effect of GEL at 168 h (*P* = 0.028, d = 0.99 [*0.08*,* 1.9*]), where passive MS was lower in GEL vs. PLA.

For concentric MS (Fig. 2B), there was a significant effect of time (*P* < 0.001, $\:{{\upeta\:}}_{\mathrm{p}}^{2}$=0.76 [*0.61*,* 0.82*]), but no significant effect of group (*P* > 0.05) or group by time interaction (*P* > 0.05). Exploratory pairwise comparisons within-group revealed increased concentric MS in PLA at 24 h (*P* < 0.001, d = 2.8 [*2.1*,* 5.6*]), 72 h (*P* < 0.001, d = 1.6 [*0.97*,* 3.3*]), and 168 h (*P* = 0.037, d = 0.69 [*0.12*,* 1.7*]), and increased concentric MS in GEL at only 24 h (*P* < 0.001, d = 2.6 [*2.1*,* 5.8*]), 72 h (*P* < 0.001, d = 2.0 [*1.5*,* 4.4*]). Between-group pairwise comparisons revealed a significant large effect of GEL at 24 h (*P* = 0.029, d = 0.97 [*0.06*,* 1.8*]), where concentric MS was lower in GEL vs. PLA.

For eccentric MS (Fig. 2C), there was a significant effect of time (*P* < 0.001, $\:{{\upeta\:}}_{\mathrm{p}}^{2}$=0.75 [*0.58*,* 0.82*]), but no significant effect of group (*P* > 0.05) or group by time interaction (*P* > 0.05). Exploratory pairwise comparisons within-group revealed increased eccentric MS in PLA at 24 h (*P* < 0.001, d = 2.9 [*2.2*,* 5.8*]), 72 h (*P* < 0.001, d = 1.5 [*0.88*,* 3.1*]), and 168 h (*P* = 0.035, d = 0.69 [*0.13*,* 17*]), and eccentric concentric MS in GEL at only 24 h (*P* < 0.001, d = 2.2 [*1.8*,* 4.8*]) and 72 h (*P* = 0.002, d = 1.4 [*1.2*,* 3.4*]). Between-group pairwise comparisons revealed a non-significant but moderate effect of GEL at 168 h (*P* = 0.086, d = 0.73 [*0.14*,* 1.6*]), where eccentric MS was lower in GEL vs. PLA.

### Muscle contractile function

**Fig. 3:**
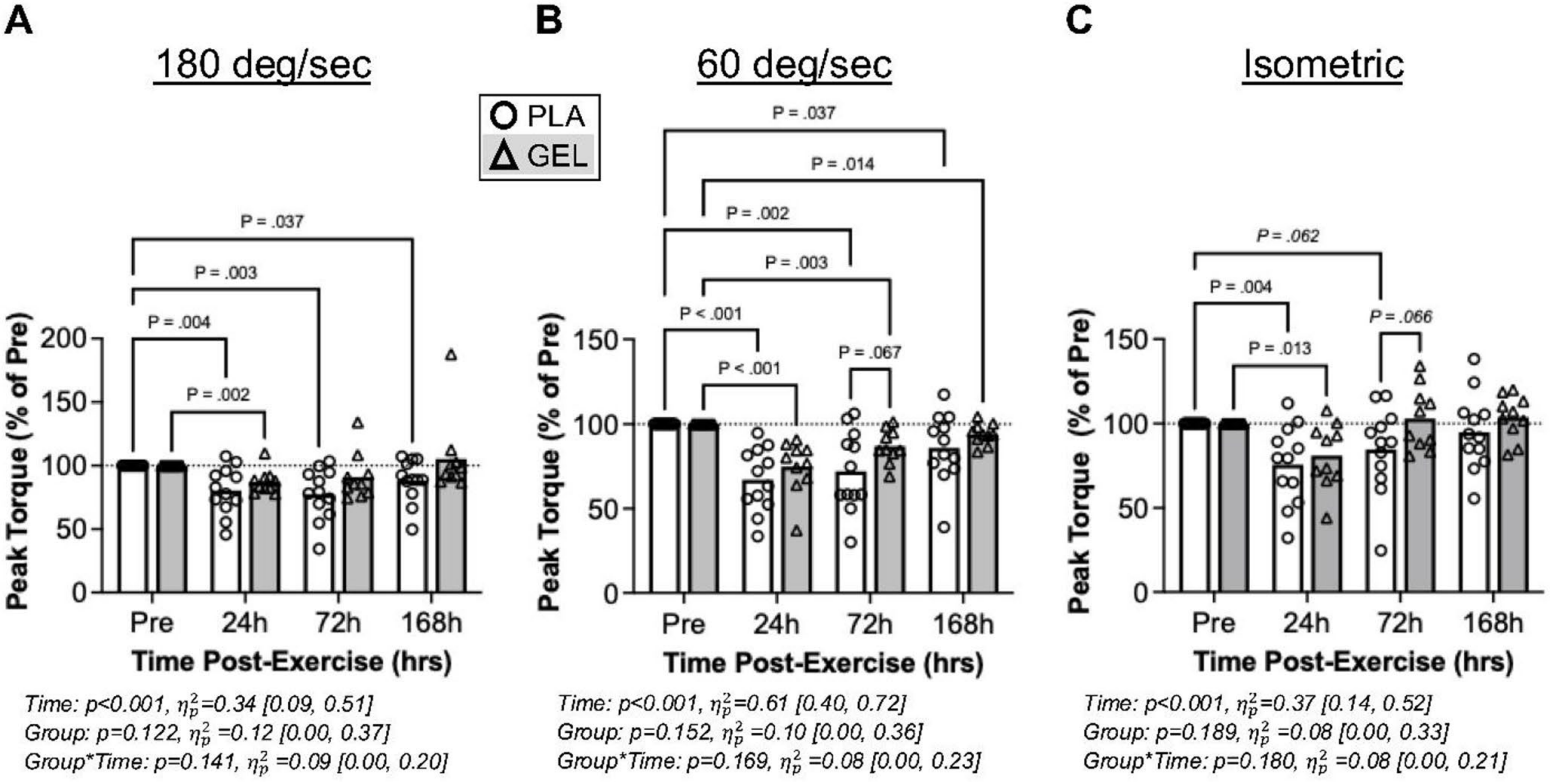
Peak contractile function prior to- and following damaging exercise. Peak isokinetic torque (PKT) of the knee extensors was assessed at two velocities: 180 deg/sec (**A**) and 60 deg/sec (**B**). Peak isometric torque (PMT) of the knee extensors (**C**) was assessed at 60 deg knee flexion. Contractile function is displayed as percentages of pre-exercise (PRE) values at 24 h, 72 h, and 168 h post-exercise. White bars and circles represent group averages and individual data for placebo (PLA; *n* = 12), respectively. Grey bars and triangles represent group averages and individual data for gelatin (GEL; *n* = 10), respectively. Dashed line denotes 100% of pre-exercise peak torque

For PKT180 (Fig. 3A), there was a significant effect of time (*P* < 0.001, $\:{{\upeta\:}}_{\mathrm{p}}^{2}$=0.34 [95% CI *0.09*,* 0.51*]), but no significant effect of group (*P* > 0.05) or group by time interaction (*P* > 0.05). Exploratory pairwise comparisons within-group revealed decreased PKT180 in PLA at 24 h (*P* = 0.004, d = 1.0 [*0.45*,* 2.5*]), 72 h (*P* = 0.003, d = 1.1 [*0.51*,* 2.6*]), and 168 h (*P* = 0.037, d = 0.69 [*0.06*,* 1.8*]), and decreased PKT180 in GEL at only 24 h (*P* = 0.002, d = 1.4 [*0.67*,* 3.1*]).

For PKT60 (Fig. 3B), there was a significant effect of time (*P* < 0.001, $\:{{\upeta\:}}_{\mathrm{p}}^{2}$=0.61 [*0.40*,* 0.72*]), but no significant effect of group (*P* > 0.05) or group by time interaction (*P* > 0.05). Exploratory pairwise comparisons within-group revealed decreased PKT60 in PLA at 24 h (*P* < 0.001, d = 1.7 [*1.2*,* 3.7*]), 72 h (*P* = 0.002, d = 1.2 [*0.60*,* 2.7*]), and 168 h (*P* = 0.037, d = 0.69 [*0.06*,* 1.8*]), and decreased PKT60 in GEL at 24 h (*P* < 0.001, d = 1.6 [*0.88*,* 3.6*]), 72 h (*P* = 0.003, d = 1.3 [*0.61*,* 3.0*]), and 168 h (*P* = 0.014, d = 0.96 [*0.27*,* 2.4*]). Between-group pairwise comparisons revealed a non-significant large effect of GEL at 72 h (*P* = 0.067, d = 0.80 [*0.09*,* 1.7*]), where PKT60 was greater in GEL vs. PLA.

For PMT (Fig. 3C), there was a significant effect of time (*P* < 0.001, $\:{{\upeta\:}}_{\mathrm{p}}^{2}$=0.37 [*0.14*,* 0.52*]), but no significant effect of group (*P* > 0.05) or group by time interaction (*P* > 0.05). Exploratory pairwise comparisons within-group revealed decreased PMT in PLA at 24 h (*P* = 0.004, d = 1.0 [*0.45*,* 2.4*]), and a non-significant moderate effect at 72 h (*P* = 0.062, d = 0.60 [*0.04*,* 1.7*]), and decreased PMT in GEL at only 24 h (*P* = 0.013, d = 0.97 [*0.27*,* 2.4*]. Between-group pairwise comparisons revealed a non-significant but large effect of GEL at 72 h (*P* = 0.066, d = 0.81 [*0.08*,* 1.7*]), where PMT was greater in GEL vs. PLA.

### Muscle morphology

Ultrasound-derived measures of MT can be found in Table 2. For MT of the VL, there was no significant effect of time (*P* > 0.05), group (*P* > 0.05), or group by time interaction (*P* > 0.05). Exploratory pairwise comparisons within-group revealed increased VL MT in PLA at 72 h (*P* = 0.014, d = 0.85 [95% CI *0.24*,* 2.1*]). Between-group pairwise comparisons revealed a significant large effect of GEL at 168 h (*P* = 0.05, d = 0.96 [*0.06*,* 1.8*]), where VL MT was lower in GEL vs. PLA. For MT of the RF, there was no significant effect of time (*P* > 0.05), group (*P* > 0.05), or group by time interaction (*P* > 0.05). Exploratory pairwise comparisons within-group revealed increased RF MT in PLA at 24 h (*P* = 0.035, d = 0.69 [*0.07*,* 1.9*]) and 72 h (*P* = 0.017, d = 0.81 [*0.20*,* 2.1*]). Between-group pairwise comparisons revealed a significant large effect of GEL at 72 h (*P* = 0.035, d = 0.95 [*0.05*,* 1.8*]), where RF MT was lower in GEL vs. PLA. For MT of the anterior VI, there was a significant effect of time (*P* = 0.007, $\:{{\upeta\:}}_{\mathrm{p}}^{2}$=0.19 [*0.02*,* 0.34*]), but no significant effect of group (*P* > 0.05), or group by time interaction (*P* > 0.05). Exploratory pairwise comparisons within-group revealed increased anterior VI MT in GEL at 24 h (*P* = 0.011, d = 1.0 [*0.31*,* 2.5*]) and 72 h (*P* = 0.05, d = 0.71 [*0.01*,* 2.0*]). Between-group pairwise comparisons revealed a significant large effect of GEL at 24 h (*P* = 0.021, d = 1.1 [*0.17*,* 2.0*]), anterior VI MT was greater in GEL vs. PLA.

### Circulating markers of collagen remodeling

**Fig. 4:**
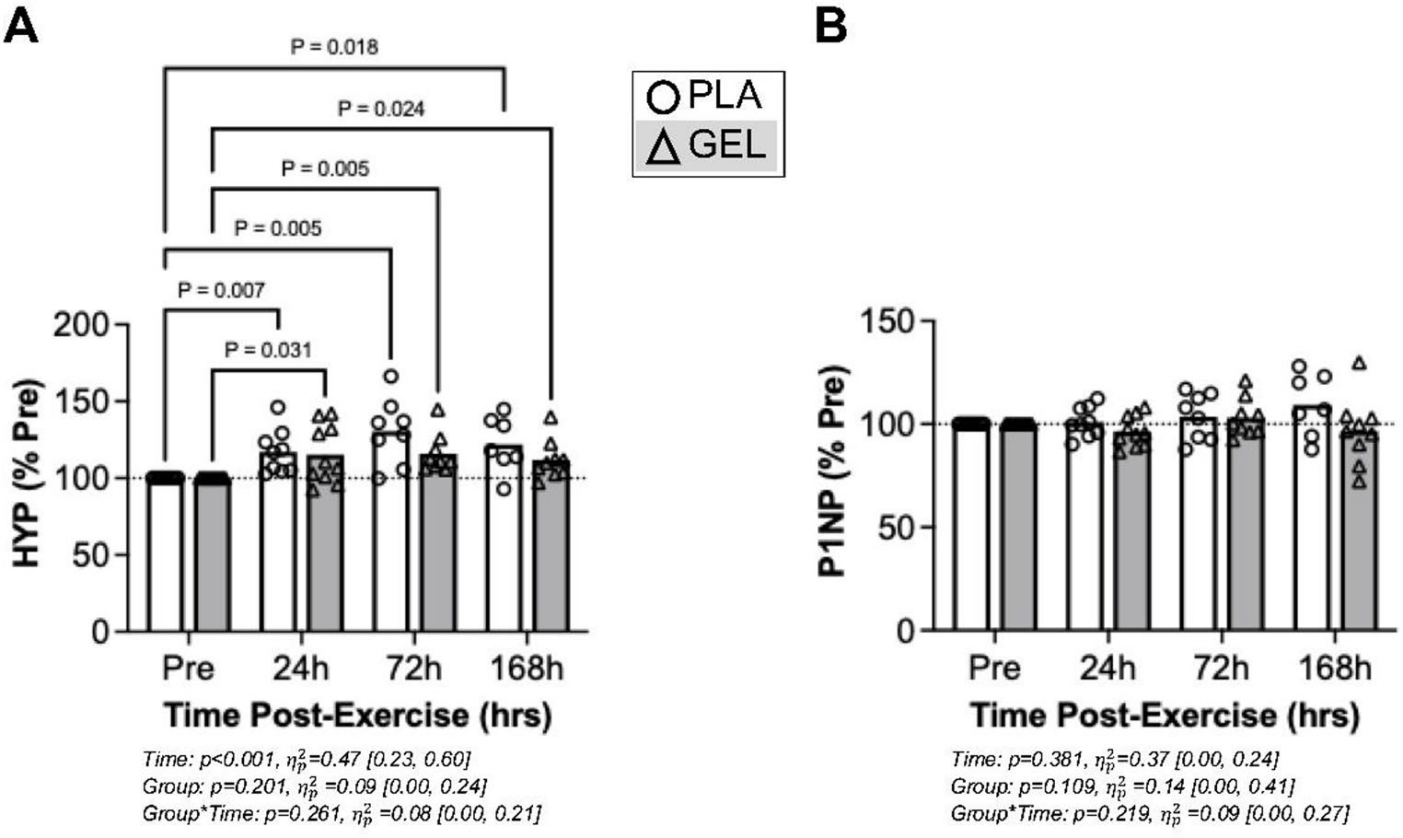
Circulating markers of collagen remodeling. Serum levels of a hydroxyproline (HYP), a marker of collagen breakdown (**A**), and procollagen 1 N-terminal peptide (P1NP), a marker of collagen synthesis (**B**) were measured prior to- and following damaging exercise. HYP and P1NP are displayed as percentages of pre-exercise (PRE) values. White bars and circles represent group averages and individual data for placebo (PLA), respectively. Grey bars and triangles represent group averages and individual data for gelatin (GEL), respectively. Dashed line denotes 100% of pre-exercise measures. Sufficient serum samples were not available for all participants at all timepoints (PLA, *n* = 7–9; GEL, *n* = 9–10)

Circulating serum levels of HYP and P1NP were assessed as indirect indicators of collagen breakdown and synthesis, respectively. For HYP (Fig. 4B), there was a significant effect of time (*P* < 0.001, $\:{{\upeta\:}}_{\mathrm{p}}^{2}$=0.47 [*0.23*,* 0.60*]), but no significant effect of group (*P* > 0.05) or group by time interaction (*P* > 0.05). Exploratory pairwise comparisons within-group revealed increased HYP in PLA at 24 h (*P* = 0.007, d = 1.2 [*0.42*,* 2.9*]), 72 h (*P* = 0.005, d = 1.4 [*0.56*,* 3.4*]), and 168 h (*P* = 0.018, d = 1.2 [*0.28*,* 3.1*]), and increased HYP in GEL at 24 h (*P* = 0.031, d = 0.81 [*0.10*,* 2.1*]), 72 h (*P* = 0.005, d = 1.3 [*0.50*,* 3.0*]), and 168 h (*P* = 0.024, d = 0.93 [*0.17*,* 2.4*]). For P1NP (Fig. 4B), there was no significant effect of time (*P* > 0.05), group (*P* > 0.05) or group by time interaction (*P* > 0.05). Exploratory pairwise comparisons within-group revealed no differences in P1NP across time in either group. Between-group pairwise comparisons revealed no differences in P1NP at any timepoint.

## Discussion

In this double-blind, placebo-controlled pilot study we examined whether gelatin supplementation influences indices of skeletal muscle recovery following an acute bout of muscle damaging eccentric exercise. We hypothesized that gelatin supplementation would expedite aspects of acute skeletal muscle recovery. Overall, the exercise protocol produced the expected soreness and impairments in contractile function, reflected by large time effects across soreness outcomes ($\:{{\upeta\:}}_{\mathrm{p}}^{2}$ = 0.56–0.76) and moderate-to-large time effects across contractile function outcomes ($\:{{\upeta\:}}_{\mathrm{p}}^{2}$ = 0.34–0.61). However, group by time interactions were not statistically significant for the primary outcomes, limiting inferences about differential recovery trajectories between supplementation groups. Therefore, the findings are best interpreted as preliminary effect estimates that can inform future, adequately powered confirmatory trials rather than as definitive evidence of treatment efficacy.

### Muscle soreness

Consistent with previous studies of EIMD (Farup et al. 2014; Hilkens et al. 2021; Brown et al. 1997b; Mackey et al. 2004; Deyhle et al. 2015; Neltner et al. 2023), the eccentric exercise protocol elicited increased ratings of MS, with large time effects ($\:{{\upeta\:}}_{\mathrm{p}}^{2}$=0.56–0.75) observed across passive, concentric, and eccentric assessments. Although the group by time interactions were not significant, exploratory pairwise comparisons suggested time-specific differences that may warrant further study. Large effects sizes for GEL were evident at 24 h with lower concentric MS (d=0.97) and 168 h with lower passive soreness (d = 0.99), suggesting GEL could influence specific phases of post-exercise MS resolution. These exploratory findings align with a prior study which showed using vitamin C enriched collagen peptides likely reduced dynamic MS following damaging exercise (Clifford et al. 2019). Interestingly, two other studies reported no effect of collagen peptide supplementation on post-exercise MS (Prowting et al. 2021; Lopez et al. 2015). However, unlike the current study and the work of Clifford et al. (2019), these studies (Prowting et al. 2021; Lopez et al. 2015) did not provide vitamin C with the collagen supplementation, suggesting the co-ingestion of vitamin C with collagen supplements may be important for reducing post-exercise MS. While no significant group by time interactions were observed, the large effects at specific timepoints in the current study coupled with evidence of supplementation reducing arthritis-related joint pain (Kumar et al. 2015; Trč and Bohmová 2011), future trials are encouraged to further investigate the impact of GEL on reducing MS.

### Muscle contractile function

Following EIMD, MS is often accompanied by transient impairments in contractile function. In agreement with prior investigations (Farup et al. 2014; Mackey and Kjaer 2017b; Brown et al. 1997b; Baumert et al. 2018; Mackey et al. 2004; Neltner et al. 2023), the eccentric exercise bout elicited significant impairments in contractile function, with large time effects ($\:{{\upeta\:}}_{\mathrm{p}}^{2}$=0.56–0.75) observed across isokinetic (PKT180, PKT60) and isometric (PMT) assessments. Similar to our findings on MS, there we no significant group by time interactions, limiting any definitive conclusions on the effect of GEL for the recovery of contractile function. However, exploratory pairwise comparisons suggest potentially meaningful effects of GEL in the intermediate recovery window (i.e. 48–72 h) that may warrant further research. Large effect sizes of GEL were observed at 72 h for PKT60 (d=0.80, *P*=0.067) and PMT (d=0.81, *P*=0.066), which is supported by a prior study on vitamin C enriched collagen peptide supplementation (Clifford et al. 2019). It is possible that GEL may accelerate the remodeling of the ECM, which is important skeletal muscle stiffness and force transmission (Gillies and Lieber 2011; Hyldahl and Hubal 2014; Mackey et al. 2011), resulting in improved force output. Mechanistically, gene expression of the major components of skeletal muscle ECM has been demonstrated to increase as early as two days following acute exercise in preclinical models (Mavropalias et al. 2023), which would align with the time course of the observed effects of GEL in the current study. Due to the pilot nature of the current study and lack of a significant group by time interaction, we are unable to definitively conclude GEL improves the recovery of contractile function. Nevertheless, the effects seen in this study could guide the selection of primary functional endpoints (i.e. low velocity, high torque/force) and timepoints (i.e. intermediate recovery, 48–72 h) in future confirmatory trials.

### Muscle morphology

In the current study, we employed noninvasive ultrasound imaging to assess muscle thickness as an indirect marker of exercise-induced swelling/edema in skeletal muscle. Previous studies have observed significant increases in MT (Nosaka and Clarkson 1996; Yu et al. 2015; Chleboun et al. 1998; Cleak and Eston 1992; Radaelli et al. 2012; Yitzchaki et al. 2020) two to five days following damaging exercise. In general, the MT outcomes did not show consistent exercise-induced changes across sites, limiting mechanistic interpretation. While several time-specific exploratory contrasts and between-group differences were observed (i.e. VL and RF MT differences at select timepoints, d = 0.69–1.1), group by time interactions were not significant and the direction of effects was not uniform across muscles/sites. Accordingly, due in part to the absence of significant exercise-induced changes in MT, we are unable to determine the impact of GEL on acute changes in muscle morphology indicative of swelling/edema. Future work employing additional imaging sites, more frequent timepoints, or complementary measures of edema/inflammation would help clarify whether GEL influences tissue morphology after damaging exercise.

### Mechanisms of gelatin supplementation

The hypothesized mechanism by which GEL supplementation might enhance post-exercise muscle recovery centers on collagen amino acid-driven collagen remodeling. As demonstrated in prior studies (Tofas et al. 2008; Mavropalias et al. 2023; Brown et al. 1997b), we observed increased HYP, a marker of collagen breakdown over time ($\:{{\upeta\:}}_{\mathrm{p}}^{2}$=0.47, *P*<0.001). However, no significant effect of group or group by time interaction were observed. In agreement with a prior study on vitamin C enriched collagen peptide supplementation (Clifford et al., 2019) , P1NP did not change over time and did not differ between groups, indicating a lack of evidence for increased collagen synthesis with the current sampling timepoints. These results weaken the mechanistic hypothesis that GEL might improve recovery via increased collagen synthesis, at least as captured by circulating P1NP and the chosen timepoints. One plausible explanation is that collagen synthesis responses may be transient and occur within hours after exercise or supplement ingestion (Lee et al. 2023; Lis and Baar 2019), whereas the present sampling focused on later recovery timepoints. These data indicate that future studies on GEL supplementation should include earlier post-exercise sampling (i.e. 1–4 h) and/or more direct measures of connective tissue remodeling. Aside from the stimulation of collagen remodeling, it is possible that GEL may exert its effects on skeletal muscle recovery through other mechanisms. Prior research has demonstrated various form of collagen supplementation to upregulate antioxidant enzymes (Liu et al. 2021), inhibit activity of metalloproteinases (MMP) 1 and 2 (Zague et al. 2011, 2018), and reduce the immune response to collagen breakdown products (Abu-Hijleh et al. 1995). Future studies of collagen supplementation are needed to determine the role these proposed mechanisms may play in the context of exercise-induced muscle damage.

### Biological sex as a moderating variable

The current sample included equal numbers of females and males, and sex-related differences in exercise-induced connective tissue remodeling are plausible (Miller et al. 2007; Kjaer 2008, PMID: 18687974). Therefore, we performed exploratory analyses including sex as a between-subject factor to evaluate potential moderation of gelatin supplementation effects across time (Supplemental Figs. 2–4). These analyses did not provide clear evidence that the time course of muscle soreness, contractile function, or circulating markers of collagen remodeling differed by sex within or between supplementation groups. Thus, given the small sex-stratified sample size of the current pilot study, a larger trial powered to test sex as a moderating variable is needed to determine whether responses differ meaningfully by biological sex. Future studies should consider hormonal contraceptive status, as was controlled for here, and menstrual cycle phase, which may impact the connective tissue response to exercise (Hansen et al. 2009; Lee et al. 2025).

### Limitations

Given the pilot nature of this study, we acknowledge that several limitations exist. First, the current sample size was not powered for small-to-moderate interaction effects, which likely contributed to wide effect-size confidence intervals and limits the ability to detect group × time differences. Second, outcomes were primarily noninvasive and functional, limiting mechanistic interpretation in the absence of direct tissue measures. Future studies employing muscle biopsies or animal models of GEL supplementation are warranted to fully understand the impact of GEL on acute exercise-induced remodeling of the ECM. Third, our study did not control for dietary intake outside of the experimental supplements, but rather, participants were instructed to maintain their normal dietary habits throughout the experimental time frame. While epidemiological data indicate levels of collagen and glycine obtained through normal diet are relatively low (Paul et al. 2019) and potentially inadequate for homeostatic requirements (Meléndez-Hevia et al. 2009), it is possible that variability in participants dietary protein and collagen-rich foods could have contributes variability to our results. Finally, although the time course selected matches common EIMD recovery windows (Brightwell et al. 2022; Mackey and Kjaer 2017b; Kjaer 2004), additional timepoints (i.e., 48 h, 96 h, 120 h) may improve resolution through which we could detect changes in recovery.

**Table 1:** Participant characteristics

Group	PLA	GEL	Between groups (*P*-values)
n (F, M)	*n* = 12 (5,7)	*n* = 10 (6,4)	
Age (yrs.)	25.8 *(8.7)*	26.1 *(4.2)*	0.897
Height (cm)	174.0 *(11.0)*	171.5 *(8.7)*	0.566
Weight (kg)	74.3 *(13.3)*	69.9 *(11.1)*	0.413
BMI (kg/m^2^)	24.4 *(3.2)*	23.7 *(2.6)*	0.571
PMT (N·m)	200.6 *(69.6)*	181.3 *(39.6)*	0.447

Data are presented as mean *(SD)* unless otherwise noted

*PLA* placebo;* GEL* gelatin;* BMI* body mass index;* PMT* pre-damaging exercise peak isometric torque

**Table 2:** Ultrasound-derived muscle thickness (% of Pre)

	PLA	GEL	ANOVA
*Vastus lateralis*			
24 h	101.2 *[98.4*,*103.9]*	99.2 *[94.9*,*103.4]*	Time: *p* = 0.481; $\:{{\upeta\:}}_{\mathrm{p}}^{2}$=0.04 [0.00,0.15]
72 h	102.0 * *[100.5*,*103.5]*	97.7 *[90.6*,*104.9]*	Group: *p* = 0.102; $\:{{\upeta\:}}_{\mathrm{p}}^{2}$=0.13 [0.00,0.39]
168 h	101.4 *[98.8*,*103.8]*	95.8 ^#^ *[90.5*,*101.1]*	Interaction: *p* = 0.09; $\:{{\upeta\:}}_{\mathrm{p}}^{2}$=0.10 [0.00,0.23]
*Lateral vastus intermedius*			
24 h	99.8 *[95.3*,*104.2]*	103.9 *[98.4*,*109.4]*	Time: *p* = 0.596; $\:{{\upeta\:}}_{\mathrm{p}}^{2}$=0.03 [0.00,0.12]
72 h	99.0 *[94.0*,*104.1]*	102.7 *[95.5*,*109.9]*	Group: *p* = 0.383; $\:{{\upeta\:}}_{\mathrm{p}}^{2}$=0.04 [0.00,0.12]
168 h	100.0 *[95.0*,*105.0]*	99.9 *[95.3*,*104.5]*	Interaction: *p* = 0.39; $\:{{\upeta\:}}_{\mathrm{p}}^{2}$=0.05 [0.00,0.15]
*Rectus femoris*			
24 h	106.0 * *[100.5*,*111.5]*	100.3 *[91.4*,*109.3]*	Time: *p* = 0.232; $\:{{\upeta\:}}_{\mathrm{p}}^{2}$=0.07 [0.00,0.20]
72 h	107.3 * *[101.6*,*113.1]*	99.4 ^#^ *[94*.1,104.7*]*	Group: *p* = 0.079; $\:{{\upeta\:}}_{\mathrm{p}}^{2}$=0.15 [0.03,0.62]
168 h	104.3 *[96.9*,*111.6]*	97.5 *[91.3*,*103.6]*	Interaction: *p* = 0.185; $\:{{\upeta\:}}_{\mathrm{p}}^{2}$=0.08 [0.00,0.19]
*Anterior vastus intermedius*			
24 h	99.3 *[92.8*,*105.9]*	110.7*^#^ *[103.1*,*118.3]*	Time: *p* = 0.007; $\:{{\upeta\:}}_{\mathrm{p}}^{2}$=0.19 [0.02,0.34]
72 h	101.8 *[92.7*,*110.9]*	107.6* *[99*.9,115.4*]*	Group: *p* = 0.105; $\:{{\upeta\:}}_{\mathrm{p}}^{2}$=0.13 [0.00,0.38]
168 h	96.5 *[89.1*,*103.9]*	98.7 *[94.8*,*102.6]*	Interaction: *p* = 0.110; $\:{{\upeta\:}}_{\mathrm{p}}^{2}$=0.10 [0.00,0.22]

Data are presented as mean* [95% confidence interval]*

*PLA* placebo; *GEL* gelatin

Exploratory pairwise comparisons: *p ≤ 0.05 vs. Pre; ^#^, *p* ≤ 0.05 vs. PLA

## Conclusions

To the best of our knowledge, this is the first study to test gelatin, a ubiquitously available and cost-effective form of supplemental collagen, as a means to impact skeletal muscle recovery from eccentric exercise. In summary, this pilot study suggests that gelatin supplementation may be associated with time-specific, moderate-to-large, estimated reductions in soreness one to seven days following damaging exercise and potentially improved torque recovery three days following damaging exercise. However, these findings were not supported by significant interaction effects and should be interpreted as preliminary. Future studies should be adequately powered to test group by time interactions, incorporate earlier biomarker sampling and/or direct tissue measurements, and prespecify primary outcomes to confirm whether gelatin supplementation meaningfully enhances recovery.

## Supplementary Information

Below is the link to the electronic supplementary material.

Supplementary Material 1

## Data Availability

Data supporting the results of the present study are available from the corresponding author upon reasonable request. Declarations.

## Abbreviations

ECM: Extracellular matrix
EIMD: Exercise-induced muscle damage
GEL: Gelatin
HYP: Hydroxyproline
MS: Muscle soreness
MT: Muscle thickness
P1NP: Pro-collagen 1 N-terminal peptide
PKT: Peak isokinetic torque
PLA: Placebo
PMT: Peak isometric torque

## References

[CR1] Abu-Hijleh MF, Habbal OA, Moqattash ST (1995) The role of the diaphragm in lymphatic absorption from the peritoneal cavity. J Anat 186(3):453–467 7559120 PMC1167005

[CR2] Baumert P, Stewart CE, Lake MJ, Drust B, Erskine RM, Consortium G-R (2018) Variations of collagen-encoding genes are associated with exercise-induced muscle damage. Physiol Genomics 50(9):691–693. 10.1152/physiolgenomics.00145.2017 29799806 10.1152/physiolgenomics.00145.2017 PMC6172608

[CR3] Berg HE, Tedner B, Tesch PA (1993) Changes in lower limb muscle cross-sectional area and tissue fluid volume after transition from standing to supine. Acta Physiol Scand 148(4):379–385. 10.1111/j.1748-1716.1993.tb09573.x 8213193 10.1111/j.1748-1716.1993.tb09573.x

[CR4] Bischof K, Stafilidis S, Bundschuh L, Oesser S, Baca A, König D (2024) Reduction in systemic muscle stress markers after exercise-induced muscle damage following concurrent training and supplementation with specific collagen peptides - a randomized controlled trial. Front Nutr 11:1384112. 10.3389/fnut.2024.1384112 38590831 10.3389/fnut.2024.1384112 PMC10999617

[CR5] Blazevich AJ, Gill ND, Zhou S (2006) Intra- and intermuscular variation in human quadriceps femoris architecture assessed in vivo. J Anat 209(3):289–310. 10.1111/j.1469-7580.2006.00619.x 16928199 10.1111/j.1469-7580.2006.00619.x PMC2100333

[CR6] Brightwell CR, Latham CM, Thomas NT, Keeble AR, Murach KA, Fry CS (2022) A glitch in the matrix: the pivotal role for extracellular matrix remodeling during muscle hypertrophy. Am J Physiol Cell Physiol 323(3):C763–C771. 10.1152/ajpcell.00200.2022 35876284 10.1152/ajpcell.00200.2022 PMC9448331

[CR7] Brown SJ, Child RB, Day SH, Donnelly AE (1997a) Exercise-induced skeletal muscle damage and adaptation following repeated bouts of eccentric muscle contractions. J Sports Sci 15(2):215–222. 10.1080/026404197367498 9258852 10.1080/026404197367498

[CR8] Brown SJ, Child RB, Day SH, Donnelly AE (1997b) Indices of skeletal muscle damage and connective tissue breakdown following eccentric muscle contractions. Eur J Appl Physiol Occup Physiol 75(4):369–374. 10.1007/s004210050174 9134370 10.1007/s004210050174

[CR9] Byrne C, Twist C, Eston R (2004) Neuromuscular function after exercise-induced muscle damage: theoretical and applied implications. Sports Med 34(1):49–69 14715039 10.2165/00007256-200434010-00005

[CR10] Cheung K, Hume P, Maxwell L (2003) Delayed onset muscle soreness: treatment strategies and performance factors. Sports Med 33(2):145–164. 10.2165/00007256-200333020-00005 12617692 10.2165/00007256-200333020-00005

[CR11] Chleboun GS, Howell JN, Conatser RR, Giesey JJ (1998) Relationship between muscle swelling and stiffness after eccentric exercise. Med Sci Sports Exerc 30(4):529–535. 10.1097/00005768-199804000-00010 9565934 10.1097/00005768-199804000-00010

[CR12] Cleak MJ, Eston RG (1992) Muscle soreness, swelling, stiffness and strength loss after intense eccentric exercise. Br J Sports Med 26(4):267–272. 10.1136/bjsm.26.4.267 1490222 10.1136/bjsm.26.4.267 PMC1479005

[CR13] Clifford T, Ventress M, Allerton DM, Stansfield S, Tang JCY, Fraser WD, Vanhoecke B, Prawitt J, Stevenson E (2019) The effects of collagen peptides on muscle damage, inflammation and bone turnover following exercise: a randomized, controlled trial. Amino Acids 51(4):691–704. 10.1007/s00726-019-02706-5 30783776 10.1007/s00726-019-02706-5

[CR14] Csapo R, Gumpenberger M, Wessner B (2020) Skeletal Muscle Extracellular Matrix - What Do We Know About Its Composition, Regulation, and Physiological Roles? A Narrative Review. Front Physiol 11:253. 10.3389/fphys.2020.00253 32265741 10.3389/fphys.2020.00253 PMC7096581

[CR15] D’Lugos AC, Luden ND, Faller JM, Akers JD, McKenzie AI, Saunders MJ (2016) Supplemental Protein during Heavy Cycling Training and Recovery Impacts Skeletal Muscle and Heart Rate Responses but Not Performance. Nutrients 8(9). 10.3390/nu8090550 10.3390/nu8090550 PMC5037535 27618091

[CR16] D’Lugos AC, Skotak NJ, Faris JJ, Thomas NT, Mazo CE, Dickinson JJ, Moore JG, Jorgensen TM, Dickinson JM (2024) Skeletal muscle architecture and aging: A comparison of ultrasound techniques and an assessment of intrarater reliability. Clin Physiol Funct Imaging. 10.1111/cpf.12882 38616358 10.1111/cpf.12882

[CR17] Daley WP, Peters SB, Larsen M (2008) Extracellular matrix dynamics in development and regenerative medicine. J Cell Sci 121(Pt 3):255–264. 10.1242/jcs.006064 18216330 10.1242/jcs.006064

[CR18] Damas F, Phillips SM, Lixandrão ME, Vechin FC, Libardi CA, Roschel H, Tricoli V, Ugrinowitsch C (2016) Early resistance training-induced increases in muscle cross-sectional area are concomitant with edema-induced muscle swelling. Eur J Appl Physiol 116(1):49–56. 10.1007/s00421-015-3243-4 26280652 10.1007/s00421-015-3243-4

[CR19] Dannecker EA, Koltyn KF (2014) Pain during and within hours after exercise in healthy adults. Sports Med 44(7):921–942. 10.1007/s40279-014-0172-z 24668291 10.1007/s40279-014-0172-z

[CR20] Deyhle MR, Gier AM, Evans KC, Eggett DL, Nelson WB, Parcell AC, Hyldahl RD (2015) Skeletal Muscle Inflammation Following Repeated Bouts of Lengthening Contractions in Humans. Front Physiol 6:424. 10.3389/fphys.2015.00424 26793125 10.3389/fphys.2015.00424 PMC4709832

[CR21] Dupuy O, Douzi W, Theurot D, Bosquet L, Dugué B (2018) An Evidence-Based Approach for Choosing Post-exercise Recovery Techniques to Reduce Markers of Muscle Damage, Soreness, Fatigue, and Inflammation: A Systematic Review With Meta-Analysis. Front Physiol 9:403. 10.3389/fphys.2018.00403 29755363 10.3389/fphys.2018.00403 PMC5932411

[CR22] Farup J, Rahbek SK, Knudsen IS, de Paoli F, Mackey AL, Vissing K (2014) Whey protein supplementation accelerates satellite cell proliferation during recovery from eccentric exercise. Amino Acids 46(11):2503–2516. 10.1007/s00726-014-1810-3 25063205 10.1007/s00726-014-1810-3

[CR23] Fry CS, Kirby TJ, Kosmac K, McCarthy JJ, Peterson CA (2017) Myogenic Progenitor Cells Control Extracellular Matrix Production by Fibroblasts during Skeletal Muscle Hypertrophy. Cell Stem Cell 20(1):56–69. 10.1016/j.stem.2016.09.010 27840022 10.1016/j.stem.2016.09.010 PMC5218963

[CR24] Gillies AR, Lieber RL (2011) Structure and function of the skeletal muscle extracellular matrix. Muscle Nerve 44(3):318–331. 10.1002/mus.22094 21949456 10.1002/mus.22094 PMC3177172

[CR25] Goodall S, Howatson G (2008) The effects of multiple cold water immersions on indices of muscle damage. J Sports Sci Med 7(2):235–241 24149455 PMC3761456

[CR26] Hansen M, Miller BF, Holm L, Doessing S, Petersen SG, Skovgaard D, Frystyk J, Flyvbjerg A, Koskinen S, Pingel J, Kjaer M, Langberg H (2009) Effect of administration of oral contraceptives in vivo on collagen synthesis in tendon and muscle connective tissue in young women. J Appl Physiol (1985) 106 (4):1435–1443. 10.1152/japplphysiol.90933.2008 10.1152/japplphysiol.90933.2008 18845777

[CR27] Hilkens L, De Bock J, Kretzers J, Kardinaal AFM, Floris-Vollenbroek EG, Scholtens PAMJ, Horstman AMH, van Loon LJC, van Dijk JW (2021) Whey protein supplementation does not accelerate recovery from a single bout of eccentric exercise. J Sports Sci 39(3):322–331. 10.1080/02640414.2020.1820184 33012216 10.1080/02640414.2020.1820184

[CR28] Hody S, Croisier JL, Bury T, Rogister B, Leprince P (2019) Eccentric Muscle Contractions: Risks and Benefits. Front Physiol 10:536. 10.3389/fphys.2019.00536 31130877 10.3389/fphys.2019.00536 PMC6510035

[CR29] Holwerda AM, van Loon LJC (2022) The impact of collagen protein ingestion on musculoskeletal connective tissue remodeling: a narrative review. Nutr Rev 80(6):1497–1514. 10.1093/nutrit/nuab083 34605901 10.1093/nutrit/nuab083 PMC9086765

[CR30] Howatson G, van Someren KA (2008) The prevention and treatment of exercise-induced muscle damage. Sports Med 38(6):483–503. 10.2165/00007256-200838060-00004 18489195 10.2165/00007256-200838060-00004

[CR31] Hyldahl RD, Hubal MJ (2014) Lengthening our perspective: morphological, cellular, and molecular responses to eccentric exercise. Muscle Nerve 49(2):155–170. 10.1002/mus.24077 24030935 10.1002/mus.24077

[CR32] Hyldahl RD, Nelson B, Xin L, Welling T, Groscost L, Hubal MJ, Chipkin S, Clarkson PM, Parcell AC (2015) Extracellular matrix remodeling and its contribution to protective adaptation following lengthening contractions in human muscle. FASEB J 29(7):2894–2904. 10.1096/fj.14-266668 25808538 10.1096/fj.14-266668

[CR33] Kehlet SN, Willumsen N, Armbrecht G, Dietzel R, Brix S, Henriksen K, Karsdal MA (2018) Age-related collagen turnover of the interstitial matrix and basement membrane: Implications of age- and sex-dependent remodeling of the extracellular matrix. PLoS ONE 13(3):e0194458. 10.1371/journal.pone.0194458 29596429 10.1371/journal.pone.0194458 PMC5875766

[CR34] Khatri M, Naughton RJ, Clifford T, Harper LD, Corr L (2021) The effects of collagen peptide supplementation on body composition, collagen synthesis, and recovery from joint injury and exercise: a systematic review. Amino Acids 53(10):1493–1506. 10.1007/s00726-021-03072-x 34491424 10.1007/s00726-021-03072-x PMC8521576

[CR35] Kjaer M (2004) Role of extracellular matrix in adaptation of tendon and skeletal muscle to mechanical loading. Physiol Rev 84(2):649–698. 10.1152/physrev.00031.2003 15044685 10.1152/physrev.00031.2003

[CR36] Krentz JR, Farthing JP (2010) Neural and morphological changes in response to a 20-day intense eccentric training protocol. Eur J Appl Physiol 110(2):333–340. 10.1007/s00421-010-1513-8 20495928 10.1007/s00421-010-1513-8

[CR37] Kumar S, Sugihara F, Suzuki K, Inoue N, Venkateswarathirukumara S (2015) A double-blind, placebo-controlled, randomised, clinical study on the effectiveness of collagen peptide on osteoarthritis. J Sci Food Agric 95(4):702–707. 10.1002/jsfa.6752 24852756 10.1002/jsfa.6752

[CR38] Kuwaba K, Kusubata M, Taga Y, Igarashi H, Nakazato K, Mizuno K (2023) Dietary collagen peptides alleviate exercise-induced muscle soreness in healthy middle-aged males: a randomized double-blinded crossover clinical trial. J Int Soc Sports Nutr 20(1):2206392. 10.1080/15502783.2023.2206392 37133292 10.1080/15502783.2023.2206392 PMC10158542

[CR39] Lee J, Tang JCY, Dutton J, Dunn R, Fraser WD, Enright K, Clark DR, Stewart CE, Erskine RM (2023) The Collagen Synthesis Response to an Acute Bout of Resistance Exercise Is Greater when Ingesting 30 g Hydrolyzed Collagen Compared with 15 g and 0 g in Resistance-Trained Young Men. J Nutr. 10.1016/j.tjnut.2023.10.030 38007183 10.1016/j.tjnut.2023.10.030 PMC11282471

[CR40] Lee J, Tang JCY, Dutton J, Dunn R, Fraser WD, Enright K, Clark DR, Stewart CE, Erskine RM (2025) Effects of resistance exercise, collagen ingestion and circulating oestrogen concentration on collagen synthesis in a female athlete: A case report. Exp Physiol 110(11):1569–1575. 10.1113/ep091897 38984642 10.1113/EP091897 PMC12575999

[CR41] Lis DM, Baar K (2019) Effects of Different Vitamin C-Enriched Collagen Derivatives on Collagen Synthesis. Int J Sport Nutr Exerc Metab 29(5):526–531. 10.1123/ijsnem.2018-0385 30859848 10.1123/ijsnem.2018-0385

[CR42] Liu X, Zu E, Chang X, Ma X, Wang Z, Song X, Li X, Yu Q, Kamei KI, Hayashi T, Mizuno K, Hattori S, Fujisaki H, Ikejima T, Wang DO (2021) Bi-phasic effect of gelatin in myogenesis and skeletal muscle regeneration. Dis Model Mech 14(12). 10.1242/dmm.049290 10.1242/dmm.049290 PMC8713995 34821368

[CR43] Lopez HL, Ziegenfuss TN, Park J (2015) Evaluation of the Effects of BioCell Collagen, a Novel Cartilage Extract, on Connective Tissue Support and Functional Recovery From Exercise. Integr Med (Encinitas) 14(3):30–38 26770145 PMC4566464

[CR47] Mackey AL, Kjaer M (2017a) The breaking and making of healthy adult human skeletal muscle in vivo. Skelet Muscle 7(1):24. 10.1186/s13395-017-0142-x 29115986 10.1186/s13395-017-0142-x PMC5688812

[CR48] Mackey AL, Kjaer M (2017b) Connective tissue regeneration in skeletal muscle after eccentric contraction-induced injury. J Appl Physiol (1985) 122 (3):533–540. 10.1152/japplphysiol.00577.2016 10.1152/japplphysiol.00577.2016 27562842

[CR46] Mackey AL, Donnelly AE, Turpeenniemi-Hujanen T, Roper HP (2004) Skeletal muscle collagen content in humans after high-force eccentric contractions. J Appl Physiol (1985) 97 (1):197–203. 10.1152/japplphysiol.01174.2003 10.1152/japplphysiol.01174.2003 14990551

[CR44] Mackey AL, Bojsen-Moller J, Qvortrup K, Langberg H, Suetta C, Kalliokoski KK, Kjaer M, Magnusson SP (2008) Evidence of skeletal muscle damage following electrically stimulated isometric muscle contractions in humans. J Appl Physiol (1985) 105(5):1620–1627. 10.1152/japplphysiol.90952.2008 18801957 10.1152/japplphysiol.90952.2008

[CR45] Mackey AL, Brandstetter S, Schjerling P, Bojsen-Moller J, Qvortrup K, Pedersen MM, Doessing S, Kjaer M, Magnusson SP, Langberg H (2011) Sequenced response of extracellular matrix deadhesion and fibrotic regulators after muscle damage is involved in protection against future injury in human skeletal muscle. FASEB J 25(6):1943–1959. 10.1096/fj.10-176487 21368102 10.1096/fj.10-176487 PMC3101036

[CR49] Mavropalias G, Boppart M, Usher KM, Grounds MD, Nosaka K, Blazevich AJ (2023) Exercise builds the scaffold of life: muscle extracellular matrix biomarker responses to physical activity, inactivity, and aging. Biol Rev Camb Philos Soc 98(2):481–519. 10.1111/brv.12916 36412213 10.1111/brv.12916

[CR50] Meléndez-Hevia E, De Paz-Lugo P, Cornish-Bowden A, Cárdenas ML (2009) A weak link in metabolism: the metabolic capacity for glycine biosynthesis does not satisfy the need for collagen synthesis. J Biosci 34(6):853–872. 10.1007/s12038-009-0100-9 20093739 10.1007/s12038-009-0100-9

[CR51] Mikkelsen UR, Schjerling P, Helmark IC, Reitelseder S, Holm L, Skovgaard D, Langberg H, Kjaer M, Heinemeier KM (2011) Local NSAID infusion does not affect protein synthesis and gene expression in human muscle after eccentric exercise. Scand J Med Sci Sports 21(5):630–644. 10.1111/j.1600-0838.2010.01170.x 20738823 10.1111/j.1600-0838.2010.01170.x

[CR52] Miller BF, Olesen JL, Hansen M, Dossing S, Crameri RM, Welling RJ, Langberg H, Flyvbjerg A, Kjaer M, Babraj JA, Smith K, Rennie MJ (2005) Coordinated collagen and muscle protein synthesis in human patella tendon and quadriceps muscle after exercise. J Physiol 567(Pt 3):1021–1033. 10.1113/jphysiol.2005.093690 16002437 10.1113/jphysiol.2005.093690 PMC1474228

[CR53] Moore DR, Phillips SM, Babraj JA, Smith K, Rennie MJ (2005) Myofibrillar and collagen protein synthesis in human skeletal muscle in young men after maximal shortening and lengthening contractions. Am J Physiol Endocrinol Metab 288(6):E1153–1159. 10.1152/ajpendo.00387.2004 15572656 10.1152/ajpendo.00387.2004

[CR54] Neltner TJ, Sahoo PK, Smith RW, Anders JPV, Arnett JE, Ortega DG, Schmidt RJ, Johnson GO, Natarajan SK, Housh TJ (2023) Effects of High-Intensity, Eccentric-Only Muscle Actions on Serum Biomarkers of Collagen Degradation and Synthesis. J Strength Cond Res 37(9):1729–1737. 10.1519/JSC.0000000000004457 37616533 10.1519/JSC.0000000000004457

[CR55] Nosaka K, Clarkson PM (1996) Changes in indicators of inflammation after eccentric exercise of the elbow flexors. Med Sci Sports Exerc 28(8):953–961. 10.1097/00005768-199608000-00003 8871903 10.1097/00005768-199608000-00003

[CR56] Nulty CD, Tang JCY, Dutton J, Dunn R, Fraser WD, Enright K, Stewart CE, Erskine RM (2024) Hydrolyzed collagen supplementation prior to resistance exercise augments collagen synthesis in a dose-response manner in resistance-trained, middle-aged men. Am J Physiol Endocrinol Metab 327(5):E668–e677. 10.1152/ajpendo.00252.2024 39259166 10.1152/ajpendo.00252.2024

[CR57] Paul C, Leser S, Oesser S (2019) Significant Amounts of Functional Collagen Peptides Can Be Incorporated in the Diet While Maintaining Indispensable Amino Acid Balance. Nutrients 11(5). 10.3390/nu11051079 10.3390/nu11051079 PMC6566836 31096622

[CR58] Peake JM, Neubauer O, Della Gatta PA, Nosaka K (2017) Muscle damage and inflammation during recovery from exercise. J Appl Physiol (1985) 122(3):559–570. 10.1152/japplphysiol.00971.2016 28035017 10.1152/japplphysiol.00971.2016

[CR59] Prowting JL, Bemben D, Black CD, Day EA, Campbell JA (2021) Effects of Collagen Peptides on Recovery Following Eccentric Exercise in Resistance-Trained Males-A Pilot Study. Int J Sport Nutr Exerc Metab 31(1):32–39. 10.1123/ijsnem.2020-0149 33186897 10.1123/ijsnem.2020-0149

[CR60] Radaelli R, Bottaro M, Wilhelm EN, Wagner DR, Pinto RS (2012) Time course of strength and echo intensity recovery after resistance exercise in women. J Strength Cond Res 26(9):2577–2584. 10.1519/JSC.0b013e31823dae96 22037095 10.1519/JSC.0b013e31823dae96

[CR61] Rozario T, DeSimone DW (2010) The extracellular matrix in development and morphogenesis: a dynamic view. Dev Biol 341(1):126–140. 10.1016/j.ydbio.2009.10.026 19854168 10.1016/j.ydbio.2009.10.026 PMC2854274

[CR62] Shaw G, Lee-Barthel A, Ross ML, Wang B, Baar K (2017) Vitamin C-enriched gelatin supplementation before intermittent activity augments collagen synthesis. Am J Clin Nutr 105(1):136–143. 10.3945/ajcn.116.138594 27852613 10.3945/ajcn.116.138594 PMC5183725

[CR63] Takala TE, Virtanen P (2000) Biochemical composition of muscle extracellular matrix: the effect of loading. Scand J Med Sci Sports 10(6):321–325. 10.1034/j.1600-0838.2000.010006321.x 11085558 10.1034/j.1600-0838.2000.010006321.x

[CR64] Thorsteinsdóttir S, Deries M, Cachaço AS, Bajanca F (2011) The extracellular matrix dimension of skeletal muscle development. Dev Biol 354(2):191–207. 10.1016/j.ydbio.2011.03.015 21420400 10.1016/j.ydbio.2011.03.015

[CR65] Tofas T, Jamurtas AZ, Fatouros I, Nikolaidis MG, Koutedakis Y, Sinouris EA, Papageorgakopoulou N, Theocharis DA (2008) Plyometric exercise increases serum indices of muscle damage and collagen breakdown. J strength conditioning Res 22(2):490–496. 10.1519/JSC.0b013e31816605a0 10.1519/JSC.0b013e31816605a0 18550965

[CR66] Trč T, Bohmová J (2011) Efficacy and tolerance of enzymatic hydrolysed collagen (EHC) vs. glucosamine sulphate (GS) in the treatment of knee osteoarthritis (KOA). Int Orthop 35(3):341–348. 10.1007/s00264-010-1010-z 20401752 10.1007/s00264-010-1010-z PMC3047656

[CR67] Yitzchaki N, Zhu WG, Kuehne TE, Vasenina E, Dankel SJ, Buckner SL (2020) An examination of changes in skeletal muscle thickness, echo intensity, strength and soreness following resistance exercise. Clin Physiol Funct Imaging 40(4):238–244. 10.1111/cpf.12630 32187417 10.1111/cpf.12630

[CR68] Yu JY, Jeong JG, Lee BH (2015) Evaluation of muscle damage using ultrasound imaging. J Phys Ther Sci 27(2):531–534. 10.1589/jpts.27.531 25729209 10.1589/jpts.27.531 PMC4339179

[CR69] Zague V, de Freitas V, da Costa Rosa M, de Castro G, Jaeger RG, Machado-Santelli GM (2011) Collagen hydrolysate intake increases skin collagen expression and suppresses matrix metalloproteinase 2 activity. J Med Food 14(6):618–624. 10.1089/jmf.2010.0085 21480801 10.1089/jmf.2010.0085

[CR70] Zague V, do Amaral JB, Rezende Teixeira P, de Oliveira Niero EL, Lauand C, Machado-Santelli GM (2018) Collagen peptides modulate the metabolism of extracellular matrix by human dermal fibroblasts derived from sun-protected and sun-exposed body sites. Cell Biol Int 42(1):95–104. 10.1002/cbin.10872 28906033 10.1002/cbin.10872

[CR71] Miller BF, Hansen M, Olesen JL, Schwarz P, Babraj JA, Smith K, Rennie MJ, Kjaer M (2007) Tendon collagen synthesis at rest and after exercise in women. J Appl Physiol 102(2): 541-546 10.1152/japplphysiol.00797.2006 10.1152/japplphysiol.00797.2006 16990502

[CR72] Kjaer M, Hansen M (2008) The mystery of female connective tissue J Appl Physiol 105(4): 1026-1027 10.1152/japplphysiol.91008.2008 10.1152/japplphysiol.91008.2008 18687974

