# Supplementary material for: Impact of gelatin supplementation on indices of skeletal muscle recovery: a pilot study
Source: Eur J Appl Physiol. 2026 Jun 2;126(8):4281–94. doi: 10.1007/s00421-026-06288-5 (PMC13451507; doi:10.1007/s00421-026-06288-5)
Supplement: Supplementary file 1 [file 421_2026_6288_MOESM1_ESM.docx]

**Journal:** European Journal of Applied Physiology

**Title: Impact of Gelatin Supplementation on Indices of Skeletal Muscle Recovery: A Pilot Study**

**Authors:** Rebecca R. Graham^2^, Brian E. Salgado^2^, Emedel Guerrero^2^, Ana P. Carrasco^2^, and Andrew C. D’Lugos^1,2^

**Institution:** ^1^Department of Kinesiology, James Madison University, Harrisonburg, VA, USA; ^2^Department of Kinesiology, California State University, Chico, Chico, CA

**Corresponding Author:** Andrew D’Lugos, PhD (dlugosac@jmu.edu)

***Supplemental Digital Content***

**Supplemental Figure 1**. Representative longitudinal plane ultrasound image of the quadriceps. Muscle thickness (MT; solid lines) was measured as the perpendicular distance between the superior and deep muscle borders [i.e., deep aponeurosis and superficial border of femur (B)] at 3 locations, corresponding to 10%, 50%, and 90% of the field of view width. SF, subcutaneous fat; RF, *rectus femoris*; VI, *vastus intermedius*; B, femur.

**Supplemental Figure 2**. Sex-stratified subjective ratings of quadriceps muscle soreness (MS) prior to- and following damaging exercise. MS was assessed during three contractile phases: static (A), concentric (B), and eccentric (C). Data represent averages ± standard deviations. Closed circles, placebo (male, n=7; female, n=5); open circles, gelatin (male, n=4; female, n=6).

**Supplemental Figure 3**. Sex-stratified peak contractile function prior to- and following damaging exercise. Peak isokinetic torque (PKT) of the knee extensors was assessed at 180 deg/sec (A) and 60 deg/sec (B). Peak isometric torque (PMT) of the knee extensors (C) was assessed at 60 deg knee flexion. Contractile function is displayed as percentages of pre-exercise (Pre) values at 24 h, 72 h, and 168 h post-exercise. Data represent averages ± standard deviations. Closed circles, placebo (male, n=7; female, n=5); open circles, gelatin (male, n=4; female, n=6).

**Supplemental Figure 4**. Sex-stratified circulating markers of collagen remodeling. Serum levels of a hydroxyproline (HYP), a marker of collagen breakdown (A), and procollagen 1 N-terminal peptide (P1NP), a marker of collagen synthesis (B) were measured prior to- and following damaging exercise. HYP and P1NP are displayed as percentages of pre-exercise (PRE) values. Data represent averages ± standard deviations. Closed circles, placebo (male, n=5; female, n=5); open circles, gelatin (male, n=4; female, n=6)
